# SAPFIR: A webserver for the identification of alternative protein features

**DOI:** 10.1186/s12859-022-04804-w

**Published:** 2022-06-24

**Authors:** Delong Zhou, Yvan Tran, Sherif Abou Elela, Michelle S. Scott

**Affiliations:** 1 Département de Microbiologie et d’infectiologie, Faculté de Médecine et des Sciences de la Santé, Université de Sherbrooke, Sherbrooke, QC J1E 4K8 Canada; 2 Département de Biochimie et Génomique Fonctionnelle, Faculté de Médecine et des Sciences de la Santé, Université de Sherbrooke, Sherbrooke, QC J1E 4K8 Canada

**Keywords:** Alternative splicing, Protein function, Protein domain, Protein domain conservation

## Abstract

**Background:**

Alternative splicing can increase the diversity of gene functions by generating multiple isoforms with different sequences and functions. However, the extent to which splicing events have functional consequences remains unclear and predicting the impact of splicing events on protein activity is limited to gene-specific analysis.

**Results:**

To accelerate the identification of functionally relevant alternative splicing events we created SAPFIR, a predictor of protein features associated with alternative splicing events. This webserver tool uses InterProScan to predict protein features such as functional domains, motifs and sites in the human and mouse genomes and link them to alternative splicing events. Alternative protein features are displayed as functions of the transcripts and splice sites. SAPFIR could be used to analyze proteins generated from a single gene or a group of genes and can directly identify alternative protein features in large sequence data sets. The accuracy and utility of SAPFIR was validated by its ability to rediscover previously validated alternative protein domains. In addition, our de novo analysis of public datasets using SAPFIR indicated that only a small portion of alternative protein domains was conserved between human and mouse, and that in human, genes involved in nervous system process, regulation of DNA-templated transcription and aging are more likely to produce isoforms missing functional domains due to alternative splicing.

**Conclusion:**

Overall SAPFIR represents a new tool for the rapid identification of functional alternative splicing events and enables the identification of cellular functions affected by a defined splicing program. SAPFIR is freely available at https://bioinfo-scottgroup.med.usherbrooke.ca/sapfir/, a website implemented in Python, with all major browsers supported. The source code is available at https://github.com/DelongZHOU/SAPFIR.

**Supplementary Information:**

The online version contains supplementary material available at 10.1186/s12859-022-04804-w.

## Background

Alternative splicing is an important cellular process which allows a single gene to produce many distinct transcripts, leading to great increase in the diversity of the proteins within a cell without increasing the number of genes [1]. It is estimated that 92–94% of human protein-coding genes undergo alternative splicing and 86% of them have a minor isoform frequency of 15% or more [2]. Alternative splicing leads to changes in the sequence of the mRNA transcript which can translate into changes in the protein product, including at the level of its localization in the cell, cellular function, stability or binding affinity [3]. Indeed, previous studies estimate that about 26% of protein domains and about 20% of localization signals are absent in some transcripts due to alternative splicing[4–6]. Alternative splicing can also change the expression of a gene by changing the stability of the mRNA or protein product [7].

In general, constitutive splicing events tend to be more conserved than alternative splicing events suggesting a role for alternative splicing in supporting species identity [8–10]. However, it remains unclear whether species-specific alternative splicing events result in species-specific alternative protein functions.

Recent advances in RNA sequencing technologies have allowed for transcriptome-wide analysis of differential splicing of mRNAs [11]. Deep sequencing of normal and diseased tissues identified thousands of splice variants underlining the potential of splicing as regulator of cell function. However, identifying the functional differences between splicing variants is limited to empirical gene-by-gene studies [3, 12]. It is unclear whether groups of genes of similar functions or genes within the same pathways are particularly prone to alternative splicing regulation. Ultimately, the association of changes in splicing profiles with specific changes in cellular function continues to be challenging [13–15].

The impact of splicing on protein function is currently being addressed by a small number of computational tools that provide information on exon or isoform functions (Table 1). These tools infer the effect of alternative splicing by predicting its impact on the presence of important protein features. For example, Exon Ontology annotates exon function by associating exons with information such as protein domains and post-translational modification sites grouped in a hierarchical structure similar to Gene Ontology [16]. On the other hand, IsoformSwitchAnalyzeR and tappAS compare splicing isoforms to determine the gain or loss of protein features including functional domains and important motifs [17, 18]. However, these tools suffer from a lack of flexible user-friendly interface, which reduces their capacity to adapt to different splicing analysis pipelines and reach a wider user base.

**Table 1:** Features of tools predicting the impact of alternative splicing on proteins functions

Tool	Interface	Resolution	Annotation	Enrichment	Species
Exon ontology	Web server	Exon	Exon ontology terms	Fixed controls	Human
IsoformSwitchAnalyzeR	R package	Isoform	Gain or loss of features	User defined controls	User defined genome
tappAS	Standalone	Isoform	Domains, motifs, sites	User defined controls	Human, mouse, Arabidopsis, fly, and maize
SAPFIR	Web server	User defined	InterProScan terms	User defined controls	Human and mouse

To facilitate the prediction of the effects of splicing events on protein function we created the **S**herbrooke **A**lternative **P**rotein **F**eature **I**dentificato**R** (SAPFIR). SAPFIR is a flexible and easy-to-use webserver that identifies alternative protein features in individual genes or lists of genomic regions. SAPFIR’s flexible parameter setup permits the analysis of any genomic region in the human and mouse genomes and can also be extended to other genomes. To demonstrate the capacity of SAPFIR, we performed genome-wide spliced domain analyses in human and mouse, showing that alternative domains display less conserved splicing patterns and in human, genes containing the most alternative domains are those involved in neurological process, transcription regulation and aging.

## Results

### SAPFIR webserver

#### 1) Annotation of alternatively included protein features

The SAPFIR webserver aims to identify and characterize alternative protein features encoded in transcripts from user-defined genes or genomic regions. To do so, SAPFIR considers all genes, transcripts, proteins as annotated in Ensembl and all protein features as predicted by InterproScan to determine those that are alternatively included. However, for a given gene, whether a protein feature is constitutive (present in all transcripts) or alternative (present only in some of the transcripts), depends on which transcripts are considered.

**Fig. 1:**
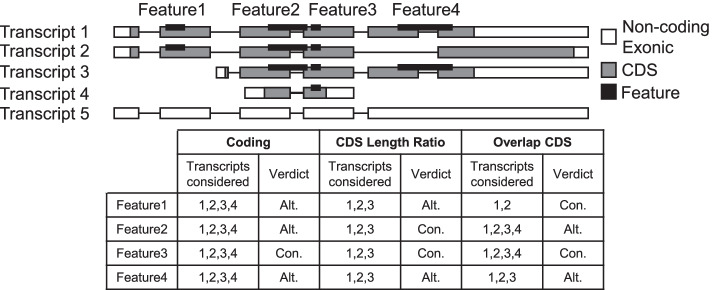
Definition of alternative protein features using an illustration of a hypothetical gene with predicted features. Whether a feature is alternative depends on which transcripts are considered. Notably, the transcripts considered by the Overlap CDS standard vary as a function of the feature in question. The CDS Length ratio is set at 50% which removes Transcript 4 from consideration. “Alt.” indicates alternative, “Con.” indicates constitutive

To provide flexibility to the user, we propose three largely independent standards to suit the needs of different studies. As illustrated in Fig. 1, isoforms can arise from alternative exons (transcripts 1 and 2), alternative transcription start sites or transcription termination sites (transcripts 2, 3 and 4), and other mechanisms. Non-coding transcripts may result from intron retention or frame shift (transcript 5). The first standard considers all coding transcripts, since including non-coding transcripts makes all predicted features alternative.

Transcripts with short coding region (CDS) pose similar challenges and are frequently observed in Ensembl annotation. To overcome this problem, we introduced a second standard where only the transcripts with long enough CDS are considered. To implement this standard, the transcript with the longest CDS in each gene is considered as the major isoform. The CDS lengths of other transcripts are compared to the major isoform to determine their ratios of CDS length. Only the transcripts whose ratios exceed a user-defined threshold are considered in this standard. The distribution of gene-wise proportion of transcripts exceeding three different thresholds (0.25, 0.50, and 0.75) are illustrated in Additional file 1: Fig. S1C. In a quarter of genes, fewer than 40% of transcripts have CDS longer than a quarter of their respective major isoform, in both human and mouse, indicating the abundance of transcripts with short CDS. Therefore, it would create significant biases if these transcripts were not excluded.

Finally, we propose a third standard of “Overlap CDS” to better identify variations caused by alternative splicing. In this standard, only transcripts where the genomic region defined by the start and end of its CDS cover entirely the genomic region defined by the start and end of a feature of interest, disregarding exon–intron boundaries during the process, are considered. These transcripts are more likely to be regulated by alternative splicing, where an alternative region is usually flanked by two constitutive regions. This standard has the particularity that transcripts considered for each feature may vary within a gene, as illustrated in Fig. 1.

#### 2) SAPFIR web interface

**Fig. 2:**
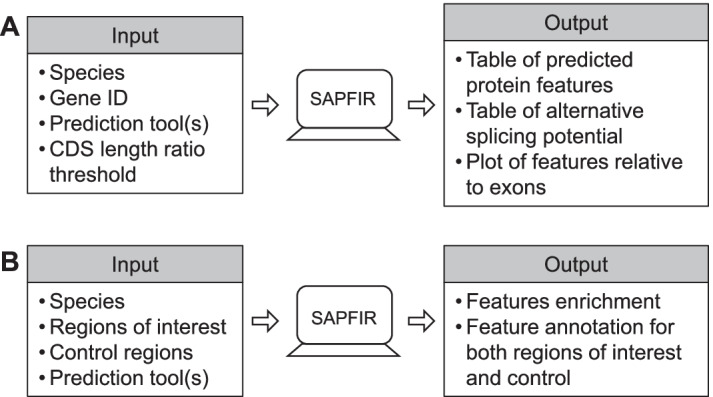
Schemes of input and output of SAPFIR webserver. Expected input information and key output of the SAPFIR webserver to annotate individual genes (Panel **A**) or lists of genomic regions (Panel **B**)

For the identification of alternative protein features in individual genes, the user starts by providing the identity of the gene of interest, defines the threshold of CDS length ratio and chooses the protein feature prediction tool(s). The result is presented as a webpage containing two downloadable and searchable tables and one graph (Fig. 2A and Additiona file 1: Fig. S2). The first table contains the protein features predicted as encoded in each transcript with their genomic position (Additional file 1: Fig. S2A). The second table indicates whether each feature is alternative or constitutive according to the standards described above (Additional file 1: Fig. S2B). Finally, the graph presents the position of the features in relation to the exons within each transcript (Additional file 1: Fig. S2C).

Rather than a single gene, users can also input a list of multiple genomic regions, which can be helpful particularly for high-throughput experiments such as RNA-seq. In this second function, SAPFIR annotates multiple genomic regions for InterProScan predicted protein features and compares the frequency of presence of the features in a list of regions of interest (referred to as the “target”) against the frequency in a list of regions of control (referred to as the “background”). A suitable target list could be splicing events found to be affected by a change in cell condition identified by differential splicing analysis of RNA-seq experiments, and the background list could be splicing events not affected during the same process.

SAPFIR can accept genomic regions that correspond to splice junctions, exons, isoforms or other user-defined regions. The user starts by providing the target and background lists, and chooses the tools used to predict protein features. The result page contains a brief summary, downloadable tables of enrichment analysis and feature annotation of the target and background lists (Fig. 2B and Additional file 1: Fig. S3). The summary contains the number of regions in both lists with the number of domains identified in total, and up to five most enriched features (Additional file 1: Fig. S3A). The enrichment table follows the same format as the table in the summary, plus links to the InterPro website for each entry. The annotated target and background tables contain the original input with predicted features in each region (Additional file 1: Fig. S3B). The SAPFIR web interface also contains a help page with hyperlinks, screenshots and figures to explain the functionality of the webserver and interpretation of results with example data (Additional file1: Fig. S4).

## Examples of use of SAPFIR

### 1) Alternative feature annotation

**Fig. 3:**
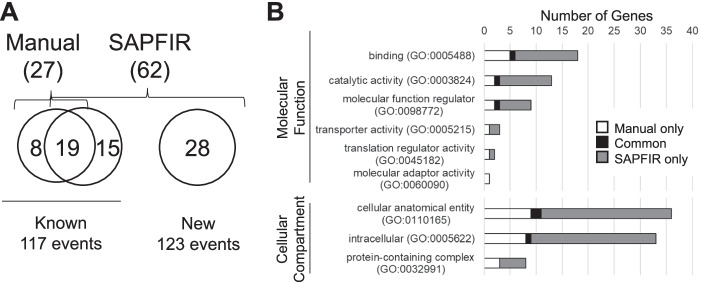
Application of SAPFIR to the characterization of alternative splicing events affected by viral infection. **A** Number of alternatively included Pfam predicted domains identified through manual curation or SAPFIR analysis. **B** Enriched Gene Ontology molecular function and cellular compartment terms related to genes with alternatively included domain. All terms presented have adjusted *p*-value < 0.05

To examine the capacity of SAPFIR to detect alternative protein features, we compared its result to that previously obtained manually for a study investigating the alternative splicing events following infection of mouse cells by the reovirus [19]. As indicated in Fig. 3A and Additional file 2: Table S1, SAPFIR identified the alternative domains in 19 out of the 27 manually annotated exons. Most domains that were not identified by SAPFIR are present in adjacent regions of the same gene (Additional file 2: Table S1). Most importantly, SAPFIR detected 15 alternative domains that were missed by the manual curation and allowed larger coverage of the alternative splicing data set resulting in the identification of 28 additional domains that were not discovered by the manual inspection (Fig. 3A). Both manual curation and SAPFIR analysis found similar protein domains within genes associated to similar molecular functions and cellular localizations (Fig. 3B, Additional file 2: Table S1), suggesting both methods have similar capacity to identify alternatively included protein features. However, usage of SAPFIR requires much less time and effort from the user, ensures consistency and allows comprehensive prediction of protein features by including other prediction tools from InterProScan, thus facilitating the functional analysis of alternative splicing.

### 2) Alternative protein domains display species-specific splicing pattern

**Fig. 4:**
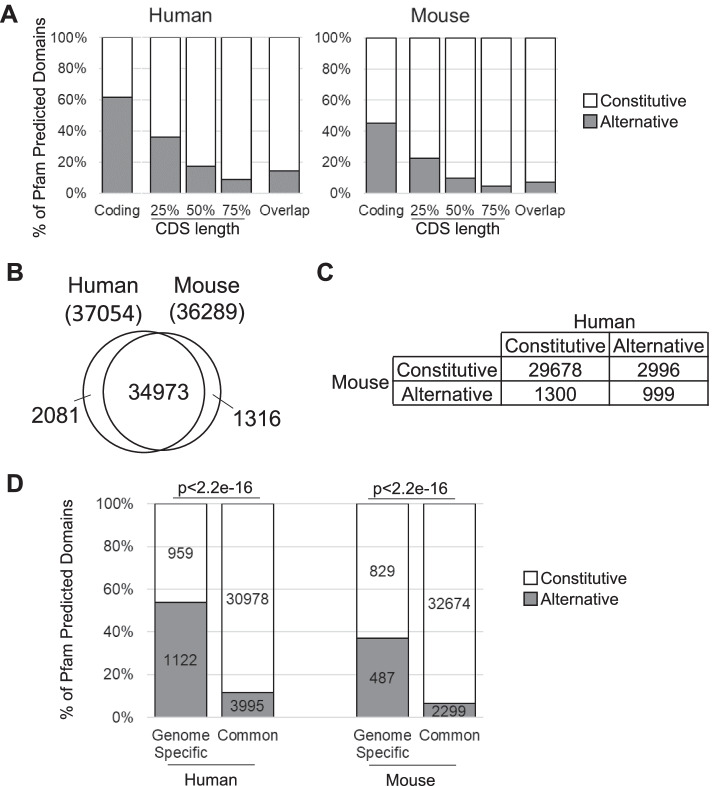
Conservation of alternatively included Pfam predicted domains. **A** Ratio of Pfam predicted domains considered as alternative in human or mouse. **B** Number of Pfam predicted domains present in homologous genes between human and mouse. **C** Number of common domains shared between homologs classified according to whether they are alternative or constitutive in either species, using the Overlap CDS standard. **D** Number of human or mouse specific domains in homologous genes that are constitutive or alternative using the Overlap CDS standard

Previous studies showed that alternative splicing events were poorly conserved between species [8]. However, it was unclear whether these species-specific alternative splicing events lead to species-specific protein function. Therefore, we compared the splicing pattern of the different human and mouse protein domains and identified those that are alternatively included in a species-specific manner. We first determined the Pfam predicted protein domains in human and mouse genomes and identified both conserved and species-specific alternative domains in human-mouse orthologs. As indicated in Fig. 4A, 60% of human domains and 45% of mouse domains were alternative based on the most relaxed standard of coding transcripts. The numbers of predicted alternative domains were reduced when only transcripts with long CDS are considered. Finally, only 14% of human domains and 7% of mouse domains were alternative using the standard most relative to alternative splicing (Overlap CDS). In general, mouse domains are less alternative than their human counterparts, which is consistent with differences in the number of transcripts per gene in the mouse and human genomes (Additional file 2: Table S2), and which also likely reflects the differences in the isoform annotation processes between the two species. Interestingly, we found that the transcription factors enriched Zinc Finger C2H2-type (IPR013087) domain was the most frequently spliced protein domain in both mouse and human genome (Additional file 2: Table S3). This indicates that while the number of alternative domains may vary, the basic functional requirement for regulating the function of this domain by alternative splicing is conserved.

To identify conserved and species-specific protein domains, we compared the domains of human and mouse orthologs as identified by Ensembl. As shown in Fig. 4B, most (> 95%) protein domains were conserved between human and mouse and most (> 90%) constitutively spliced domains in one genome were also constitutively spliced in the other. In contrast, only 25% (999 out of 3997) of alternative human domains were alternative in mouse, and only 43% (999 out of 2299) of alternative mouse domains were alternative in human (Fig. 4C). Furthermore, 54% (1122 out of 2081) of human-specific domains found in the orthologs were alternative (Fig. 4D), much higher than the common domains (11%, *p* < 2.2e-16). A similar result was observed in mouse (Fig. 4D). Accordingly, we conclude that the splicing pattern of alternative domains is in general less conserved across genomes than that of constitutively spliced domains.

### 3) The alternative splicing potential of protein domains is linked to domain and gene functions

**Fig. 5:**
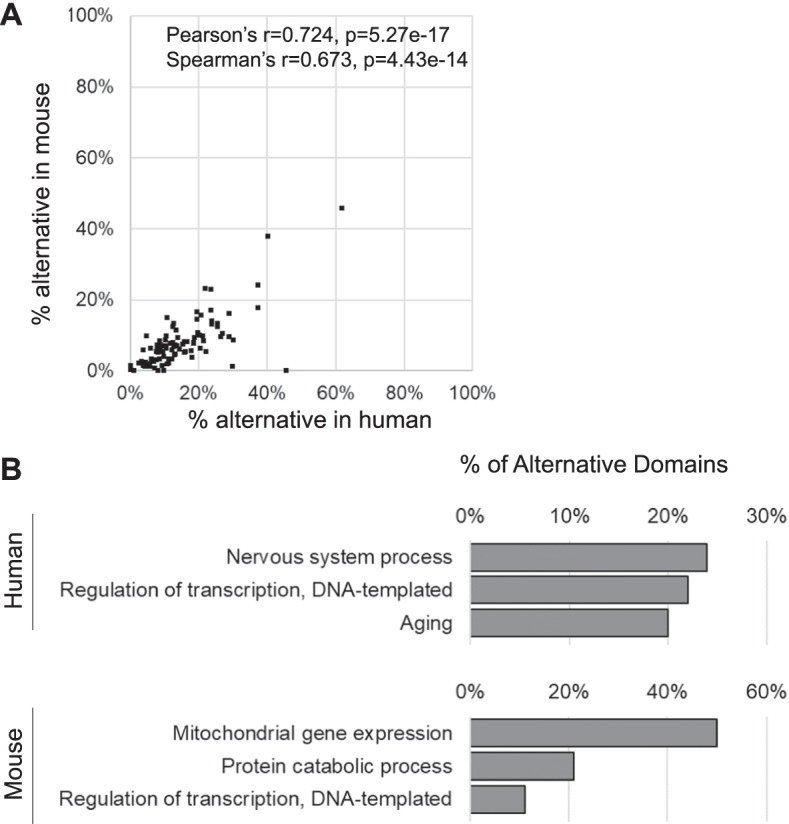
Domains and cellular processes that are most likely regulated by alternative splicing. **A** Comparison of splicing potential of common domains between human and mouse. Pearson correlation coefficient and Spearman’s rank correlation coefficient are indicated as well as their respective *p* values. Only domains found at least 50 times in both genomes are considered, resulting in a total of 97 domains. **B** Cellular processes which contain the most alternative domains (predicted by Pfam) in human and mouse, using the Overlap CDS standard

Interestingly, we found that common domains between human and mouse display similar splicing potential as shown through the high level of correlation but that not all protein domains display the same level of alternative splicing potential (Fig. 5A). For example, we found that the protein–protein interaction domains are highly alternative in general. Three out of the five most alternative domains in both human and mouse genomes were Ankyrin repeat (IPR002110), Nebulin repeat (IPR000900), and IQ motif EF-hand binding site (IPR000048), which are among the most widely distributed protein–protein interaction motifs (Additional file 2: Table S4). On the other hand, the least alternative domains in both genomes were receptor or inhibitor domains (Additional file 2: Table S4). We conclude that the protein functional domains do not all have the same potential for alternative splicing and that certain domain functions like protein–protein interaction are particularly targeted for splicing dependent regulation.

Since domains with different functions display different splicing potential, we asked whether proteins with different functions may display different propensity for regulation through alternative splicing. As indicated in Fig. 5B, we found that indeed genes with different functions have different levels of alternatively spliced domains. For example, in the human genome the processes carried out by neurological organs contained the highest percentage of alternative domains, followed by the regulation of transcription and aging (Fig. 5B and Additional file 2: Table S5). Interestingly, while the regulation of transcription remained among the processes with most alternative domains in mouse, mitochondrial gene expression and protein catabolic processes were the most alternative domains in mouse instead of neurological processes or aging in human (Fig. 5B). The biological process differences in alternative domains reflect the number of domains present in each group of genes, and not changes in the alternative splicing frequency of any given domain. Indeed, the alternative splicing frequency of each domain type mostly remained similar between the genes associated to these terms and the genomic average (Additional file 2: Table S6). We conclude that highly alternative domains are enriched in groups of genes involved in same biological processes in a species-specific manner.

## Conclusion and discussion

In this study, we implemented SAPFIR, a flexible and user-friendly webtool to facilitate the study of functional consequences of alternative splicing in human and mouse by linking variations in mRNA sequences to those in functional protein features (Figs. 1 and 2). Compared to existing tools, SAPFIR is more flexible in parameter setting and can be extended to other species, thus providing better capacity to adapt to various functional studies of alternative splicing [16–18].

SAPFIR is especially helpful to predict the functional impact of changes in splicing profile detected by RNA-seq by performing functional annotation for a list of genomic regions (Figs. 2 and 3). The splicing changes that affect the presence of important protein features are more likely to change the function of the proteins, thus providing a priority list for downstream validation and directions for further studies.

We find that although protein domains are largely conserved between human and mouse, the splicing patterns of alternative domains are less conserved than those of constitutive domains (Fig. 4), similar to what was observed on the exon level [8–10, 20, 21]. This finding reemphasizes the importance of genome specificity in functional analysis of alternative splicing. In addition, species-specific domains are particularly more alternative, in accordance with previous suggestions of alternative splicing as a source of protein functional innovation and adaptive benefit [20, 22].

In human, genes with most alternative domains are related to neurological process, transcription regulation and aging (Fig. 5B). The most frequent domains found in each group are consistent with their respective functions (for example transmembrane ion transport for neurological process), and these domains are not particularly more alternative compared to the rest of genes in the genome (Fig. 5B, Additional file 2: Table S5 and S6). Previously it was shown that neural alternative splicing events regulate protein–protein interactions [23, 24]. Our data suggest that neural alternative splicing could also regulate protein functions related to transmembrane ion transport (Additional file 2: Table S6). The alternative splicing patterns of several genes were associated with aging and age-related diseases, however the global functional impact remained unclear [25, 26]. Here our data suggest that alternative splicing may regulate protein functions related to cellular structure (Additional file 2: Table S6).

SAPFIR currently relies on InterProScan to predict protein function from mRNA sequences, thus could benefit from improvements of the InterProScan algorithms or better tools to predict protein functions in general. The quality of SAPFIR analysis also depends on the quality of upstream differential splicing analysis. A robust differential splicing analysis with high precision and accuracy will increase the number and quality of alternatively spliced events and produce a better description of functional impact of the changes in alternative splicing profile. The functionality of SAPFIR can be further extended by incorporating additional interesting features including protein–protein interaction sites, pre-mature stop codons, isoform expression or splicing profiles from cell lines and tissues.

## Methods

### Construction and use of SAPFIR webserver

#### Construction of SAPFIR database

To identify alternative protein domains in both human and mouse, we started by building a database housing all required data. Human (GRCh38 release 103) and mouse (GRCm39 release 103) genome annotations (.gtf files) and protein sequences (.fa files) were obtained from Ensembl [27]. Protein features predicted by InterProScan v 5.40–77.0 [28]. The APPRIS database was used to identify the principal isoform of protein-coding genes [29]. These data were combined into a local sqlite3 database as shown in Additional file 1: Fig. S1A. Numbers of coding genes, their transcripts, exons, coding regions (CDS) and predicted protein features are listed in Additional file 2: Table S2. The number of protein features predicted by each member database of InterProScan are further detailed in Additional file 2: Table S2. On average, 12.4 features were predicted in a human transcript and 14.4 features predicted in a mouse transcript. Data processing was performed with Python v 3.9.5, pandas v 1.2.5 and sqlite3 v 2.6.0.

#### Web server implementation

The web server was constructed with Python v3.9.5 and Django v3.2.3, with two main functions: single gene annotation and multiple genomic regions annotation, as described below.

#### Single gene annotation

The first function of SAPFIR is to identify alternatively included protein features in a single gene. To do so, the gene, transcripts, exons and domain features were retrieved from the database and presented as the first table in the output page. The genomic coordinates of predicted features are compared to each other to identify the common domains among transcripts. Features with overlapping genomic positions are considered as common. The features are then examined to determine whether they are present in all candidate transcripts, where the candidate transcripts consist of either (A) all coding transcripts, (B) transcripts whose CDS are longer than a user-defined fraction of the longest CDS in the gene, or (C) transcripts whose CDS cover the genomic region corresponding to the feature (referred to as the Overlap CDS standard). The result is displayed as the second table in the output page. Finally, the positions of protein features relative to the exons are plotted for all transcripts of the gene, and plotted as a graph using Python v3.9.5. The final graph is displayed in the output page following the two tables described above.

#### Multiple genomic regions annotation

The second function of SAPFIR is to identify enriched protein features in a list of genomic regions of interest (referred to as the “target”) compared to a list of control genomic regions (referred to as the “background”). To do this, the two lists are compared with the database to annotate the predicted features that overlap with these regions using pybedtools, a Python implementation of Bedtools [30, 31]. The number of overlaps for each feature is then compared between the two lists and a chi-square test is performed using scipy v1.7.0 to determine whether a feature is more frequent in either list [32]. The p-value of the chi-square test is then adjusted by the Benjamini–Hochberg Procedure (Additional file 1: Fig. S1B). A fold change of enrichment is calculated as the ratio of the frequency in the target list over the frequency in target and background combined, to avoid division by zero errors.

Genomic regions from previously published data were provided as example data [19, 33].

### Identification of homologous genes between human and mouse

Human and mouse homologs were retrieved through Ensembl BioMart web session along with percentage identity both from human to mouse gene and mouse to human gene. For each query gene, the target gene with the highest query identity was considered the best hit. Pairs of genes that are reciprocal best hits were considered homologs to each other. This process identified 18,213 pairs of homologs between human and mouse, which are listed in the Additional file 2: Table S7.

### Identification of alternative domains associated with Gene Ontology (GO) terms

A list of 143 GO Slim generic terms was retrieved from the Gene Ontology project [34, 35]. BioMart was used to retrieve genes associated with each of these GO terms from the Ensembl database [36, 37]. Protein domains were predicted in these genes using Pfam, a prediction tool covering many common protein domains [38]. Predicted domains were then compared between transcripts of each gene to determine whether they were constitutive or alternative using the Overlap CDS standard as described above. Chi-square tests were performed using scipy v1.7.0 to determine whether Pfam predicted domains were more alternative in genes associated with a GO term than the genomic average. The p-values of the chi-square test were then adjusted by the Benjamini–Hochberg Procedure when appropriate.

## Supplementary Information

**Additional file1: Fig. S1 **(related to Figure 1) Schematic representation of the alternative protein domain identification pipeline. (A) Pipeline used to produce SAPFIR database. (B) Pipeline used to perform the enrichment test (C) Distribution of the percent transcripts retained at different thresholds of genes CDS length ratio. The box ranges from the first quartile to the third quartile of the distribution with a line across the box indicating the median. The whiskers extend to the minimal values in each distribution. **Fig. S2 **related to Figure 2. Example of the gene features display. Screen shots of the different tables showing features of human RBFOX2 gene produced using Pfam as InterPro member database and a CDS length ratio threshold of 50%. (A) Table describing the predicted features (e.g. protein domain) and their associated transcripts and genomic positions. In the Transcript ID column, two transcripts are tagged with ** to indicate that they are considered as major isoforms of the gene by the APPRIS database. (B) Table describing the alternative potential of the protein features (e.g. alternative or constitutive). (C) Graphic representation of the RBFOX2 alternative protein features predicted by Pfam (partial). Boxes represent exons and lines represent introns, drawn to scale. White and blue portions of boxes illustrate respectively non-coding and coding sections of exons. Protein features are drawn as thick colored lines overlapping exons that encode them and their intervening introns. Protein features are annotated with a label indicating their InterPro identifier or prediction signature. Within this panel, if a protein feature is present more than once, all instances are represented by the same color. **Fig. S3 **related to Figure 2 Screenshot of features enrichment analysis using previously annotated examples. **Fig. S4 **Screenshot of SAPFIR’s help page with hyperlinks and figures to explain the functionality of the webserver and interpretation of results with example data.

**Additional file2: Table S1 **Manual and SAPFIR annotation of splicing events affected by viral infection in mouse. **Table S2** Number of coding genes, their transcripts, exons and predicted features. **Table S3** Most frequent domains found in human and mouse. **Table S4** Most and least alternatively included Pfam predicted domains in human and mouse. **Table S5 **Percentage of alternative domains associated to GO slim terms.Adjuted p-values indicate the siginificance of whether the percentage of alternative domains associated to GO terms is different from the genomic average. **Table S6** Domains associated to GO terms identified in Figure 5B. **Table S7 **Homolog genes in human and mouse genome

## Data Availability

SAPFIR is available at https://bioinfo-scottgroup.med.usherbrooke.ca/sapfir/. Source code to reproduce SAPFIR for an annotated genome is available at https://github.com/DelongZHOU/SAPFIR.

## Abbreviations

CDS: Coding regions
GO: Gene ontology
SAPFIR: Sherbrooke alternative protein feature identificator
