# Supplementary material for: SAPFIR: A webserver for the identification of alternative protein features
Source: BMC Bioinformatics. 2022 Jun 24;23:250. doi: 10.1186/s12859-022-04804-w (PMC9229502; doi:10.1186/s12859-022-04804-w)
Supplement: Supplementary file 1 — Additional file1: Fig. S1 (related to Figure 1) Schematic representation of the alternative protein domain identification pipeline. (A) Pipeline used to produce SAPFIR database. (B) Pipeline used to perform the enrichment test (C) Distribution of the percent transcripts retained at different thresholds of genes CDS length ratio. The box ranges from the first quartile to the third quartile of the distribution with a line across the box indicating the median. The whiskers extend to the minimal values in each distribution. Fig. S2 related to Figure 2. Example of the gene features display. Screen shots of the different tables showing features of human RBFOX2 gene produced using Pfam as InterPro member database and a CDS length ratio threshold of 50%. (A) Table describing the predicted features (e.g. protein domain) and their associated transcripts and genomic positions. In the Transcript ID column, two transcripts are tagged with ** to indicate that they are considered as major isoforms of the gene by the APPRIS database. (B) Table describing the alternative potential of the protein features (e.g. alternative or constitutive). (C) Graphic representation of the RBFOX2 alternative protein features predicted by Pfam (partial). Boxes represent exons and lines represent introns, drawn to scale. White and blue portions of boxes illustrate respectively non-coding and coding sections of exons. Protein features are drawn as thick colored lines overlapping exons that encode them and their intervening introns. Protein features are annotated with a label indicating their InterPro identifier or prediction signature. Within this panel, if a protein feature is present more than once, all instances are represented by the same color. Fig. S3 related to Figure 2 Screenshot of features enrichment analysis using previously annotated examples. Fig. S4 Screenshot of SAPFIR’s help page with hyperlinks and figures to explain the functionality of the webserver and interpretation of results with [file 12859_2022_4804_MOESM1_ESM.pptx]

## Slide 1
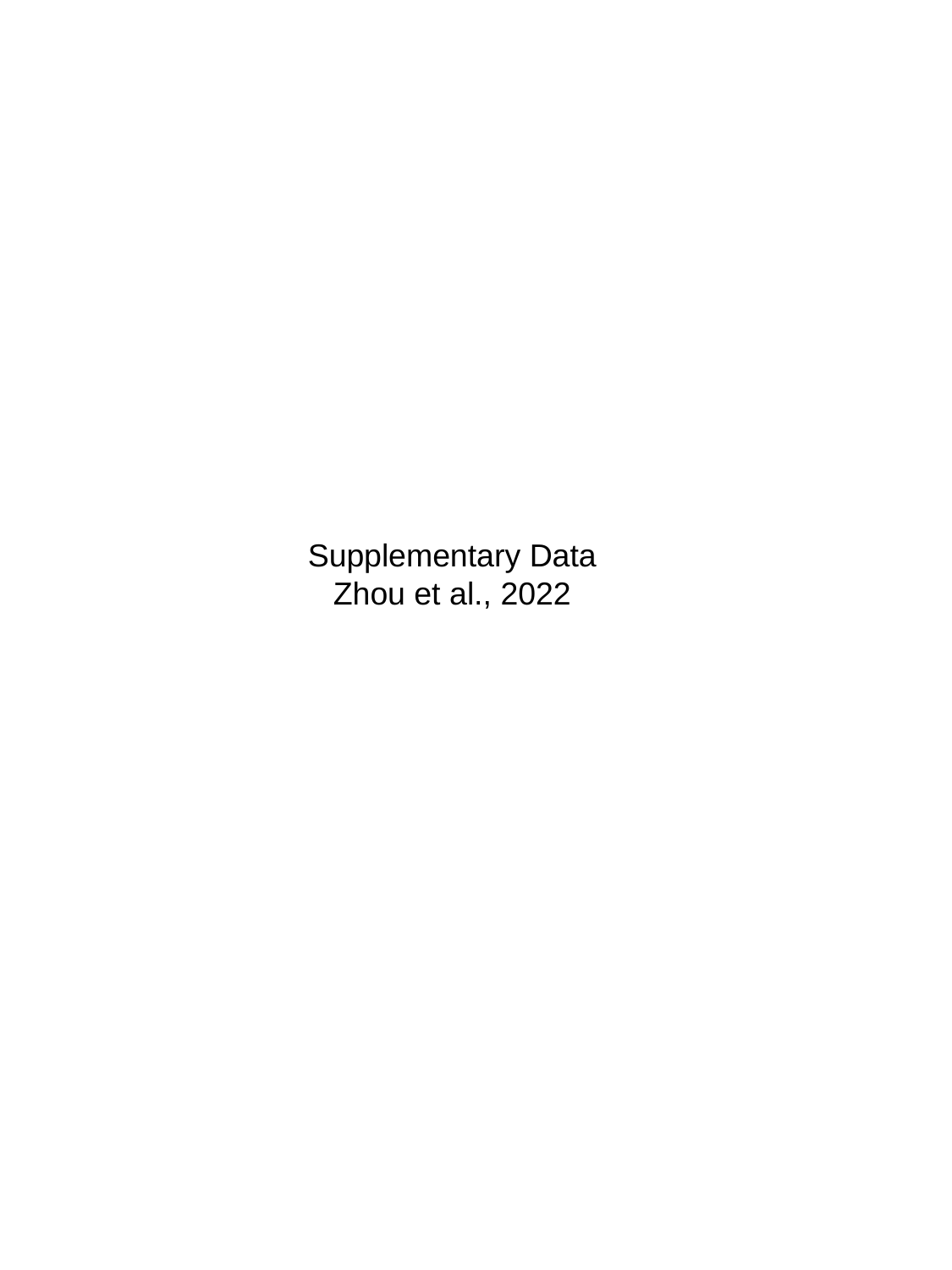

Supplementary Data
Zhou et al., 2022

## Slide 2
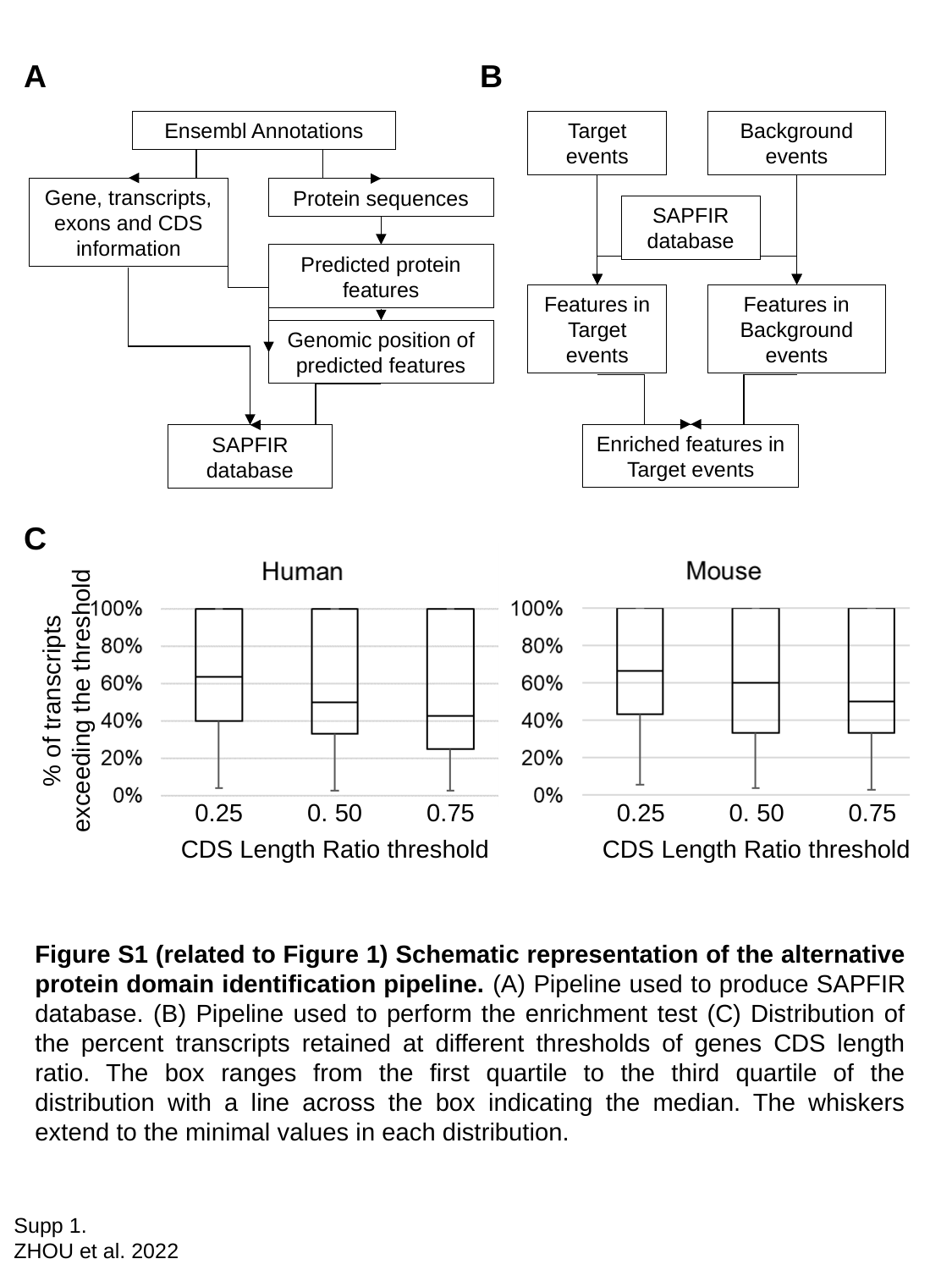

A
B
Target events
Background events
SAPFIR database
Features in Target events
Features in Background events
Enriched features in Target events
Ensembl Annotations
Gene, transcripts, exons and CDS information
Protein sequences
Predicted protein features
Genomic position of predicted features
SAPFIR database
C
% of transcripts exceeding the threshold
CDS Length Ratio threshold
CDS Length Ratio threshold
0.25
0. 50
0.75
0.25
0. 50
0.75
Figure S1 (related to Figure 1) Schematic representation of the alternative protein domain identification pipeline. (A) Pipeline used to produce SAPFIR database. (B) Pipeline used to perform the enrichment test (C) Distribution of the percent transcripts retained at different thresholds of genes CDS length ratio. The box ranges from the first quartile to the third quartile of the distribution with a line across the box indicating the median. The whiskers extend to the minimal values in each distribution.
Supp 1.
ZHOU et al. 2022

## Slide 3
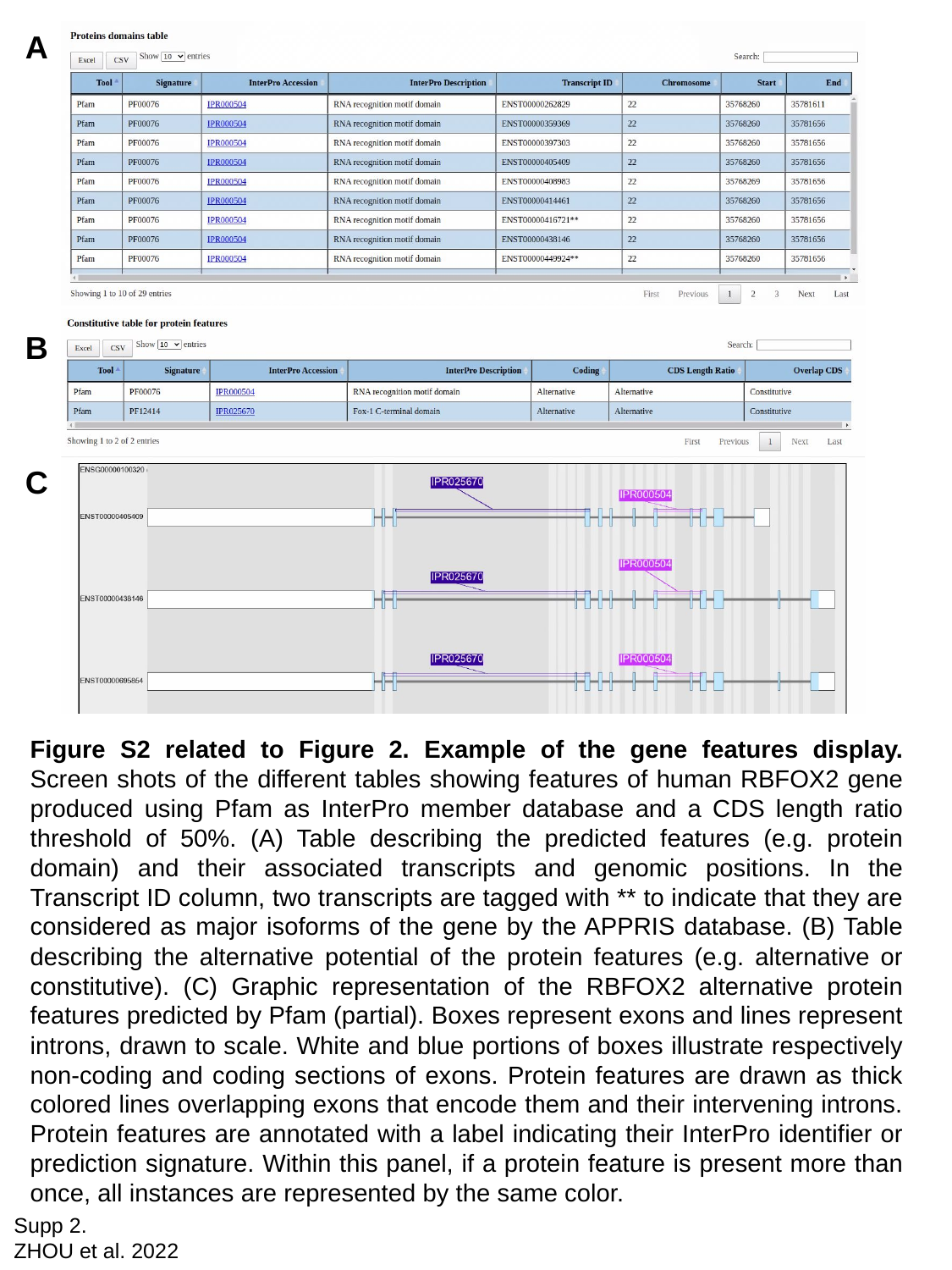

A
B
C
Figure S2 related to Figure 2. Example of the gene features display. Screen shots of the different tables showing features of human RBFOX2 gene produced using Pfam as InterPro member database and a CDS length ratio threshold of 50%. (A) Table describing the predicted features (e.g. protein domain) and their associated transcripts and genomic positions. In the Transcript ID column, two transcripts are tagged with ** to indicate that they are considered as major isoforms of the gene by the APPRIS database. (B) Table describing the alternative potential of the protein features (e.g. alternative or constitutive). (C) Graphic representation of the RBFOX2 alternative protein features predicted by Pfam (partial). Boxes represent exons and lines represent introns, drawn to scale. White and blue portions of boxes illustrate respectively non-coding and coding sections of exons. Protein features are drawn as thick colored lines overlapping exons that encode them and their intervening introns. Protein features are annotated with a label indicating their InterPro identifier or prediction signature. Within this panel, if a protein feature is present more than once, all instances are represented by the same color.
Supp 2.
ZHOU et al. 2022

## Slide 4
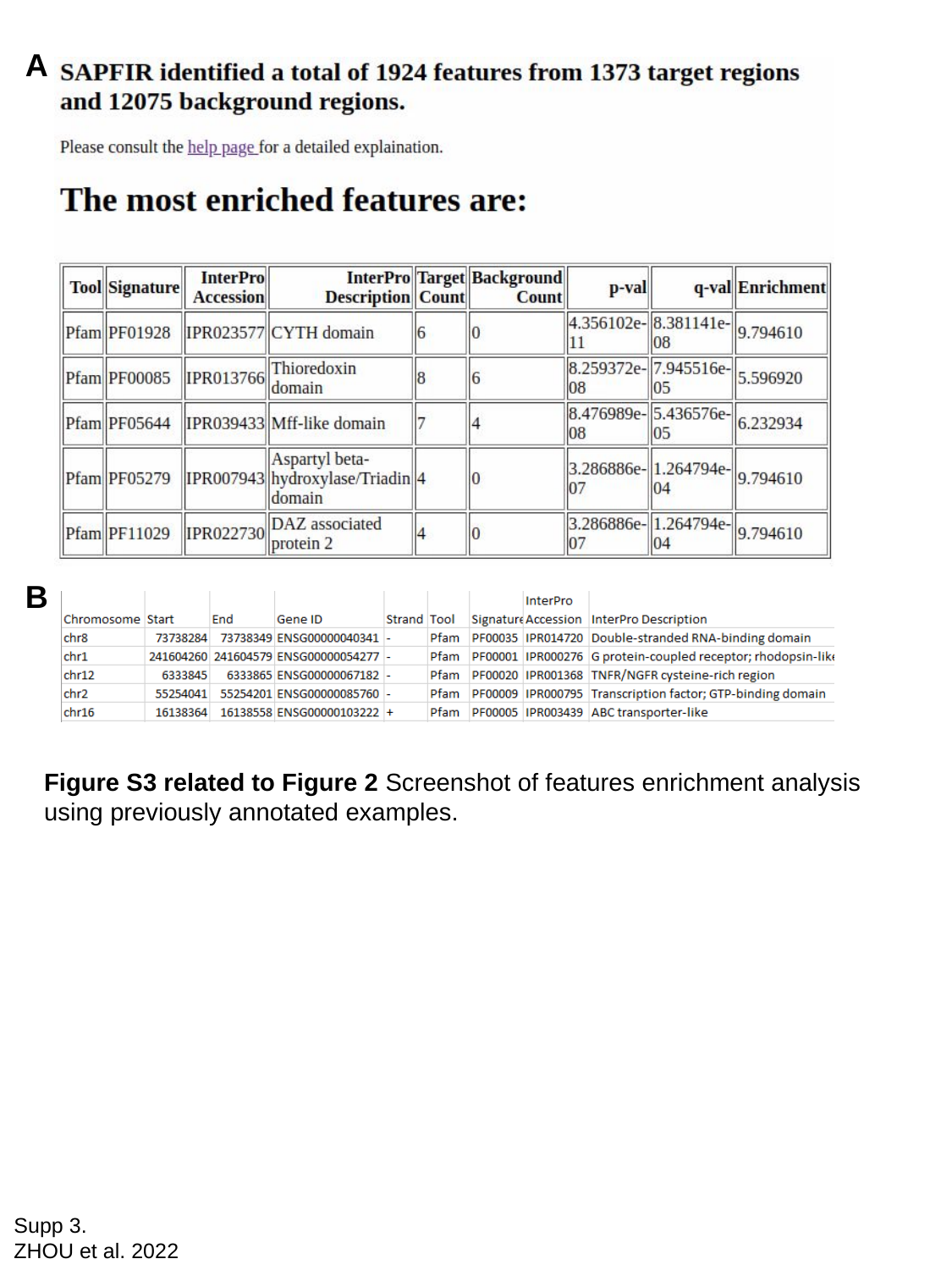

A
B
Figure S3 related to Figure 2 Screenshot of features enrichment analysis using previously annotated examples.
Supp 3.
ZHOU et al. 2022

## Slide 5
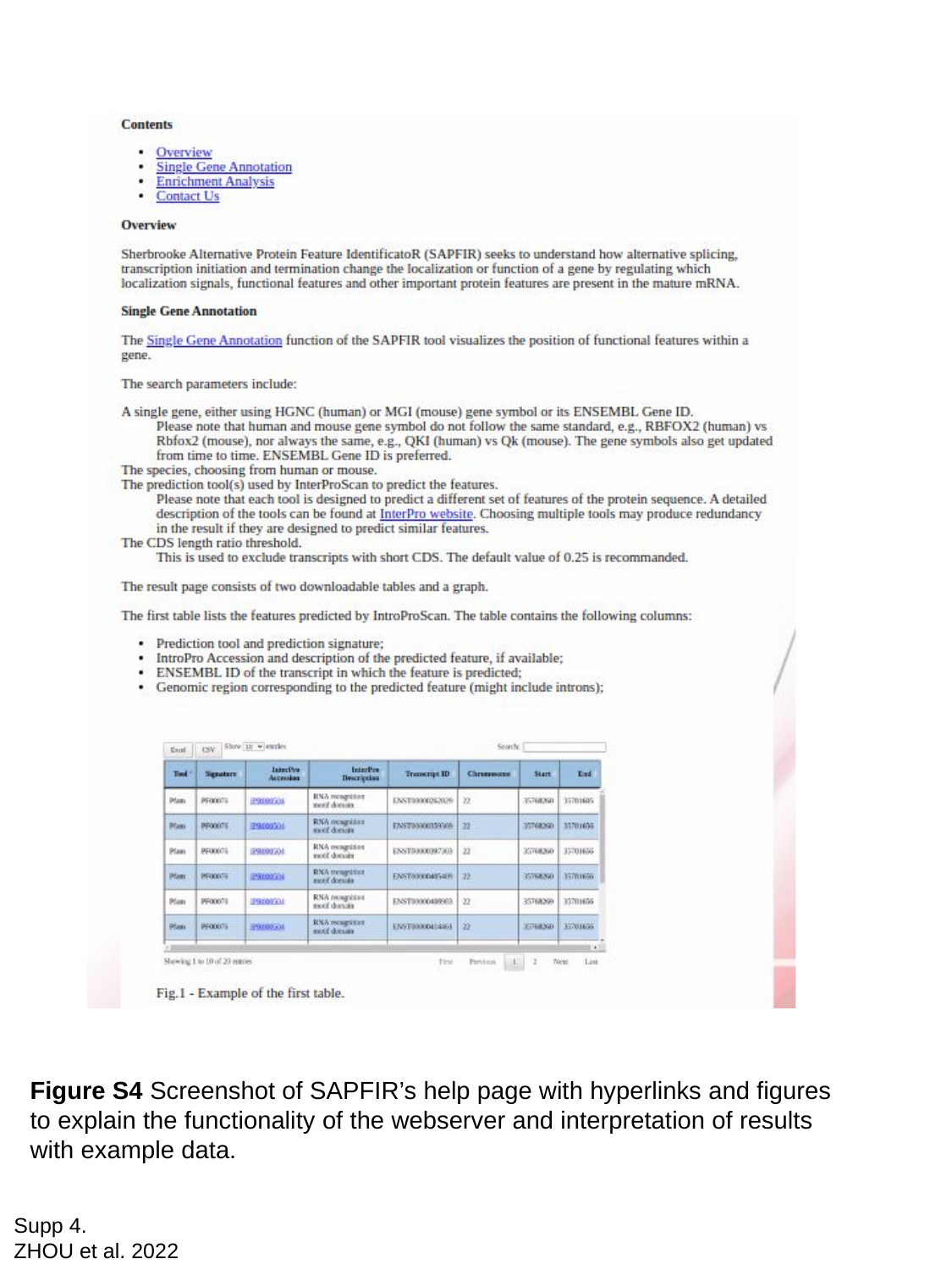

Figure S4 Screenshot of SAPFIR’s help page with hyperlinks and figures to explain the functionality of the webserver and interpretation of results with example data.
Supp 4.
ZHOU et al. 2022
